# Supplementary material for: ImPaqT, a Golden Gate-based immunological toolkit for zebrafish transgenesis
Source: eLife. 2026 Jul 29;14:RP104182. doi: 10.7554/eLife.104182 (PMC13421555; doi:10.7554/eLife.104182)
Supplement: Supplementary file 1. — (A) Sequence of runx1 +23 including PaqCI sites, O1 and O2. (B) Sequence of tdStayGold including PaqCI sites, O2 and O3. (C) Sequence of LNGFR including PaqCI sites, O2 and O3. (D) Sequence of rac2WT including PaqCI sites, overhang sequence CTCG (O3B) and O4. (E) Sequence of rac2D57N including PaqCI sites, O3B and O4. (F) Sequence of 3E polyA-gRNA (Control):U6 including PaqCI sites, overhang sequence CGTA (O3C) and O4. (G) Sequence of 3E polyA-gRNA (GFP):U6 including PaqCI sites, O3C and O4. [file elife-104182-supp1.docx]

**ImPaqT - A Golden Gate-based Toolkit for Zebrafish Transgenesis**

Saskia Hurst, Christiane Dimmler, Mark R. Cronan

**Supplementary File 1: Sequences of insertion constructs purchased as dsDNA fragments.**

**A**

TTTCCCCACCTGCCCTTTTCCGGGGTGGGAGGTGTAAGTTCCACCCCCACCCTTCCTGACACGCTCCTGAACCCTGGCCACTGCACCTGGCTAGGTTCTCACTTCTCTGGGAAGCATCTAGAAACAGGACCTCTCACCCACCCCTCCCGGCTGGGCTCTAGGGTGGGGCCCTCACTACCTCTTTTCTTCTCAAAGAGCCTGGGATGCTGACAGCCTCAGATGGAGGCATCCTGTTTGTCGAAAAATAAACCGGCAGTTGAAGCCGGGTTGCAAGAGCGAGAAAACCGCAGGCCTGCGCGCCACTGATAACGTGGGCAGCTTGCTTTTGCAGCAGTTCCTAGCTGCAGCGGCCCTGTGAAGGCCTGTGTCACCGCCTCCCTTCCTGTCTCCTCCTCACACCATCCCTCCATCGCTCCTTGCTGGCTCTACCAGCCACTTGCTGGACCCTTCAGCCACTGGGACCATTGCTTTCCATAAAAAACTCCTTAGTGCAGTGCAGAGCTGATCAGAGGGTAGCAGGGAACCCTGACGCCTGATGGGTTGCTGACACCTGAGATCCACTAGTCCAATCTGCTCAGAGAGGACAGAGTGGGCAGGAGCCAGCATTGGGTATATAAAGCTGAGCAGGGTCAGTTGCTTCTTACGTTTGCTTCTGATTCTGTTGTGTTGACTTGCAACCTCAGAAACAGACATCCAAACCTTGCAGGTGGGGTTT

**B**

TTTCCCCACCTGCCCTTCAAACATGGGAGAGGAGCTGTTCACAGGAGTGGTGATGGCTAGTACACCATTTAAATTTCAACTTAAAGGAACCATCAATGGCAAATCGTTTACCGTTGAAGGCGAAGGTGAAGGAAATTCACATGAAGGTTCTCATAAAGGAAAATATGTTTGTACAAGTGGAAAACTACCGATGTCATGGGCAGCACTTGGAACATCCTTTGGTTATGGAATGAAATATTATACCAAATATCCTAGTGGACTGAAGAACTGGTTTCATGAAGTAATGCCTGAAGGCTTTACCTACGATCGTCATATTCAATATAAAGGCGATGGGAGTATCCATGCAAAACACCAACACTTTATGAAAAATGGGACTTATCACAACATTGTTGAATTTACTGGTCAGGATTTTAAAGAAAATAGTCCAGTCTTAACTGGAGATATGAATGTCTCATTACCGAATGAAGTTCAACATATACCCAGAGATGATGGAGTAGAATGCCCAGTGACCTTGCTTTATCCTTTATTATCGGATAAATCAAAATGCGTTGAGGCTCACCAAAATACAATCTGCAAGCCTCTTCATAATCAACCAGCACCTGATGTCCCATATCACTGGATTCGTAAACAATACACACAAAGCAAAGATGATACCGAGGAACGTGATCATATTTGTCAATCAGAGACTCTCGAAGCACACTTAGGAAACCCTTGGCACGAGCCTAGCGCTAGCGCTGTGAGTGCCGGCGGTTCTGCAGGCGGGTCTGCAGGAGGTTCAGCTGGTGGTAGTGCAGGCGGCTCTGCAGGCGGTGGGGAAGAACTGTTTACCGGCGTTGTTATGGCATCCACTCCTTTCAAGTTCCAGCTCAAGGGTACAATAAACGGAAAGTCCTTCACAGTAGAGGGTGAAGGCGAGGGTAACAGTCACGAGGGGTCCCACAAGGGCAAGTACGTCTGCACCAGCGGTAAGCTGCCAATGTCTTGGGCTGCGCTGGGTACGAGTTTCGGGTACGGGATGAAGTACTACACAAAGTACCCATCTGGCCTTAAGAATTGGTTCCACGAGGTCATGCCAGAGGGGTTCACGTATGACAGGCACATCCAGTACAAAGGGGACGGCTCAATACACGCGAAGCATCAGCATTTCATGAAGAACGGTACATACCATAATATAGTAGAGTTCACAGGACAAGACTTCAAGGAGAACAGCCCTGTGCTAACAGGGGACATGAACGTATCCCTGCCTAACGAGGTACAGCACATCCCTCGAGACGACGGCGTGGAGTGTCCCGTTACGCTACTGTACCCACTGCTTTCCGACAAGTCTAAGTGTGTGGAAGCCCATCAGAACACGATTTGTAAACCCCTGCACAACCAGCCCGCCCCCGACGTGCCCTACCATTGGATCCGAAAGCAGTATACCCAGTCTAAGGACGACACTGAAGAGCGCGACCACATCTGCCAGAGCGAAACACTAGAGGCCCATTTGGGCAATCCCTGGCATGAACCGTCCGCCAGTGCAGTCTAGGCTACCTTGCAGGTGGGGTTT

**C**

TTTCCCCACCTGCCCTTCAAACATGGGAGCTGGAGCTACAGGCAGAGCTATGGATGGACCTAGACTGCTGCTGCTGCTGCTGCTGGGTGTGAGCCTGGGAGGAGCTAAGGAAGCTTGTCCCACAGGACTGTATACTCACTCTGGAGAGTGTTGTAAGGCCTGTAACCTGGGAGAGGGCGTGGCTCAGCCATGTGGAGCTAATCAGACAGTGTGCGAGCCCTGTCTGGACTCTGTGACTTTCAGTGACGTGGTGAGTGCTACCGAGCCCTGTAAACCATGCACCGAGTGCGTGGGACTGCAGTCTATGAGTGCTCCATGCGTGGAGGCTGACGACGCCGTGTGCAGATGCGCTTACGGATATTACCAGGACGAGACAACAGGAAGATGCGAGGCTTGTAGAGTCTGCGAAGCAGGAAGCGGACTGGTGTTCAGCTGTCAAGATAAACAGAACACAGTGTGCGAGGAGTGCCCAGACGGAACCTATAGCGACGAGGCCAACCATGTCGATCCTTGCCTCCCTTGTACAGTGTGCGAGGACACTGAGAGACAGCTGCGAGAGTGTACCAGATGGGCAGACGCTGAGTGTGAGGAGATTCCAGGAAGATGGATCACAAGAAGTACACCTCCAGAGGGCTCCGACAGCACAGCCCCATCCACACAGGAGCCTGAAGCTCCACCTGAGCAGGACCTGATCGCTTCCACTGTGGCAGGAGTCGTGACAACTGTGATGGGCAGCTCTCAGCCCGTGGTGACACGTGGCACAACCGACAACCTGATCCCAGTGTATTGCTCCATCCTGGCCGCTGTGGTGGTGGGCCTGGTGGCTTACATCGCTTTCAAACGCTGAGCTACCTTGCAGGTGGGGTT

**D**

ACTGACTGCACCTGCCCTTCTCGATGCAAGCAATAAAGTGTGTGGTGGTCGGAGATGGAGCTGTGGGAAAGACCTGTCTTCTCATCAGCTACACTACCAATGCGTTCCCCGGGGAGTACATTCCCACAGTGTTTGATAACTACTCTGCAAATGTAATGGTGGATAGCAAACCAGTCAACCTGGGACTCTGGGATACAGCCGGACAGGAAGATTATGACAGACTGCGGCCACTCTCCTACCCGCAGACGGATGTGTTTCTTATCTGTTTCTCTTTGGTGAGCCCAGCATCATTCGAAAATGTCAGAGCCAAGTGGTACCCAGAGGTGAGGCATCACTGCCCTTCCACTCCAATTATCCTGGTTGGCACCAAGCTTGACTTGAGAGATGAGAAGGAGACCATCGAGAAGCTGAAGGAGAAGAAACTGGCACCGATCACTTACCCACAGGGTCTCGCATTGGCCAAAGAAATAGATGCAGTAAAATACCTGGAGTGTTCGGCCCTCACTCAGAGAGGGCTAAAAACAGTGTTTGATGAGGCGATTCGCGCTGTGCTCTGCCCACAGCCCACCAAGGTCAAGAAGAAGGGCTGCGTGATGCTCTAAAGGACCTTGCAGGTGACTGACTG

**E**

ACTGACTGCACCTGCCCTTCTCGATGCAAGCAATAAAGTGTGTGGTGGTCGGAGATGGAGCTGTGGGAAAGACCTGTCTTCTCATCAGCTACACTACCAATGCGTTCCCCGGGGAGTACATTCCCACAGTGTTTGATAACTACTCTGCAAATGTAATGGTGGATAGCAAACCAGTCAACCTGGGACTCTGGAATACAGCCGGACAGGAAGATTATGACAGACTGCGGCCACTCTCCTACCCGCAGACGGATGTGTTTCTTATCTGTTTCTCTTTGGTGAGCCCAGCATCATTCGAAAATGTCAGAGCCAAGTGGTACCCAGAGGTGAGGCATCACTGCCCTTCCACTCCAATTATCCTGGTTGGCACCAAGCTTGACTTGAGAGATGAGAAGGAGACCATCGAGAAGCTGAAGGAGAAGAAACTGGCACCGATCACTTACCCACAGGGTCTCGCATTGGCCAAAGAAATAGATGCAGTAAAATACCTGGAGTGTTCGGCCCTCACTCAGAGAGGGCTAAAAACAGTGTTTGATGAGGCGATTCGCGCTGTGCTCTGCCCACAGCCCACCAAGGTCAAGAAGAAGGGCTGCGTGATGCTCTAAAGGACCTTGCAGGTGACTGACTG

**F**

AGCGCCCAATACGCAAACCGCCTCTCCCCGCGCGTTGGCCGATTCATTAATGCAGCTGGCACGACAGGTTTCCCGACTGGAAAGCGGGCAGTGAGCGCAACGCAATTAATGTGAGTTAGCTCACTCATTAGGCACCCCAGGCTTTACACTTTATGCTTCCGGCTCGTATGTTGTGTGGAATTGTGAGCGGATAACAATTTCACACAGGAAACAGCTATGACCATGATTACGCCAAGCTATTTAGGTGACACTATAGAATACTCAAGCTATGCATCAAGCTTGGTACCGAGCTCGGATCCACTAGTAACGGCCGCCAGTGTGCTGGAATTCGCCCTTCACCTGCCCTTCGTAAGATCTATAATTCACTGGCCGTCGTTTTACGGTACCATCGATGATGATCCAGACATGATAAGATACATTGATGAGTTTGGACAAACCACAACTAGAATGCAGTGAAAAAAATGCTTTATTTGTGAAATTTGTGATGCTATTGCTTTATTTGTAACCATTATAAGCTGCAATAAACAAGTTAACAACAACAATTGCATTCATTTTATGTTTCAGGTTCAGGGGGAGGTGTGGGAGGTTTTTTAAAGCAAGTAAAACCTCTACAAATGTGGTATGGCTGATTATGATCCTCTAGATCGAGGTCTCTGACTAAAAAAGCACCGACTCGGTGCCACTTTTTCAAGTTGATAACGGACTAGCCTTATTTAAACTTGCTATGCTGTTTCCAGCATAGCTCTTAAACAGAGACGGTCGACAGTCTGCAGTGTCGTCTCTCGAACCAAGAGCTGGAGGGAGAGCTATATATACCAGGGACTTCTGGGTATGTTTTTGGGAGGTGGTGAGTGACTAAACCACTTATTCAGCTCCCTTAGATCAAGTCTGACCCTATATCATGGTGACATAAACCTGCAAACTGATAAAACCTGAAGGATCTCAAATCCAGAGTTTGTGTGAGGGATTACCGTGGTAATTTTCAAACTGTAAAGCATATGCAAAATTATCTGGTCTTGGCTTGAGTGATTGGGTGTCTCGGTGTGATGCAGGGACGTTTTCAGTGACGTGTCCTTCTCCCTCCCCCACAGGCATGCGCAGAACATTTCCCCCCTCCTTGAAGACCAGAACAAAAGACGCCGAGAGCAGGAAACTCGTCTTACTGAATGACCGAGGCTGGAGAAAGTCGACCCTAGGAGGACCTTGCAGGTGAAGGGCGAATTCTGCAGATATCCATCACACTGGCGGCCGCTCGAGCATGCATCTAGAGGGCCCAATTCGCCCTATAGTGAGTCGTATTACAATTCACTGGCCGTCGTTTTACAACGTCGTGACTGGGAAAACCCTGGCGTTACCCAACTTAATCGCCTTGCAGCACATCCCCCTTTCGCCAGCTGGCGTAATAGCGAAGAGGCCCGCACCGATCGCCCTTCCCAACAGTTGCGCAGCCTATACGTACGGCAGTTTAAGGTTTACACCTATAAAAGAGAGAGCCGTTATCGTCTGTTTGTGGATGTACAGAGTGATATTATTGACACGCCGGGGCGACGGATGGTGATCCCCCTGGCCAGTGCACGTCTGCTGTCAGATAAAGTCTCCCGTGAACTTTACCCGGTGGTGCATATCGGGGATGAAAGCTGGCGCATGATGACCACCGATATGGCCAGTGTGCCGGTCTCCGTTATCGGGGAAGAAGTGGCTGATCTCAGCCACCGCGAAAATGACATCAAAAACGCCATTAACCTGATGTTCTGGGGAATATAAATGTCAGGCATGAGATTATCAAAAAGGATCTTCACCTAGATCCTTTTCACGTAGAAAGCCAGTCCGCAGAAACGGTGCTGACCCCGGATGAATGTCAGCTACTGGGCTATCTGGACAAGGGAAAACGCAAGCGCAAAGAGAAAGCAGGTAGCTTGCAGTGGGCTTACATGGCGATAGCTAGACTGGGCGGTTTTATGGACAGCAAGCGAACCGGAATTGCCAGCTGGGGCGCCCTCTGGTAAGGTTGGGAAGCCCTGCAAAGTAAACTGGATGGCTTTCTCGCCGCCAAGGATCTGATGGCGCAGGGGATCAAGCTCTGATCAAGAGACAGGATGAGGATCGTTTCGCATGATTGAACAAGATGGATTGCACGCAGGTTCTCCGGCCGCTTGGGTGGAGAGGCTATTCGGCTATGACTGGGCACAACAGACAATCGGCTGCTCTGATGCCGCCGTGTTCCGGCTGTCAGCGCAGGGGCGCCCGGTTCTTTTTGTCAAGACCGACCTGTCCGGTGCCCTGAATGAACTGCAAGACGAGGCAGCGCGGCTATCGTGGCTGGCCACGACGGGCGTTCCTTGCGCAGCTGTGCTCGACGTTGTCACTGAAGCGGGAAGGGACTGGCTGCTATTGGGCGAAGTGCCGGGGCAGGATCTCCTGTCATCTCACCTTGCTCCTGCCGAGAAAGTATCCATCATGGCTGATGCAATGCGGCGGCTGCATACGCTTGATCCGGCTACCTGCCCATTCGACCACCAAGCGAAACATCGCATCGAGCGAGCACGTACTCGGATGGAAGCCGGTCTTGTCGATCAGGATGATCTGGACGAAGAGCATCAGGGGCTCGCGCCAGCCGAACTGTTCGCCAGGCTCAAGGCGAGCATGCCCGACGGCGAGGATCTCGTCGTGACCCATGGCGATGCCTGCTTGCCGAATATCATGGTGGAAAATGGCCGCTTTTCTGGATTCATCGACTGTGGCCGGCTGGGTGTGGCGGACCGCTATCAGGACATAGCGTTGGCTACCCGTGATATTGCTGAAGAGCTTGGCGGCGAATGGGCTGACCGCTTCCTCGTGCTTTACGGTATCGCCGCTCCCGATTCGCAGCGCATCGCCTTCTATCGCCTTCTTGACGAGTTCTTCTGAATTATTAACGCTTACAATTTCCTGATGCGGTATTTTCTCCTTACGCATCTGTGCGGTATTTCACACCGCATACAGGTGGCACTTTTCGGGGAAATGTGCGCGGAACCCCTATTTGTTTATTTTTCTAAATACATTCAAATATGTATCCGCTCATGAGACAATAACCCTGATAAATGCTTCAATAATAGCACGTGAGGAGGGCCACCATGGCCAAGTTGACCAGTGCCGTTCCGGTGCTCACCGCGCGCGACGTCGCCGGAGCGGTCGAGTTCTGGACCGACCGGCTCGGGTTCTCCCGGGACTTCGTGGAGGACGACTTCGCCGGTGTGGTCCGGGACGACGTGACCCTGTTCATCAGCGCGGTCCAGGACCAGGTGGTGCCGGACAACACCCTGGCCTGGGTGTGGGTGCGCGGCCTGGACGAGCTGTACGCCGAGTGGTCGGAGGTCGTGTCCACGAACTTCCGGGACGCCTCCGGGCCGGCCATGACCGAGATCGGCGAGCAGCCGTGGGGGCGGGAGTTCGCCCTGCGCGACCCGGCCGGCAACTGCGTGCACTTCGTGGCCGAGGAGCAGGACTGACACGTGCTAAAACTTCATTTTTAATTTAAAAGGATCTAGGTGAAGATCCTTTTTGATAATCTCATGACCAAAATCCCTTAACGTGAGTTTTCGTTCCACTGAGCGTCAGACCCCGTAGAAAAGATCAAAGGATCTTCTTGAGATCCTTTTTTTCTGCGCGTAATCTGCTGCTTGCAAACAAAAAAACCACCGCTACCAGCGGTGGTTTGTTTGCCGGATCAAGAGCTACCAACTCTTTTTCCGAAGGTAACTGGCTTCAGCAGAGCGCAGATACCAAATACTGTCCTTCTAGTGTAGCCGTAGTTAGGCCACCACTTCAAGAACTCTGTAGCACCGCCTACATACCTCGCTCTGCTAATCCTGTTACCAGTGGCTGCTGCCAGTGGCGATAAGTCGTGTCTTACCGGGTTGGACTCAAGACGATAGTTACCGGATAAGGCGCAGCGGTCGGGCTGAACGGGGGGTTCGTGCACACAGCCCAGCTTGGAGCGAACGACCTACACCGAACTGAGATACCTACAGCGTGAGCTATGAGAAAGCGCCACGCTTCCCGAAGGGAGAAAGGCGGACAGGTATCCGGTAAGCGGCAGGGTCGGAACAGGAGAGCGCACGAGGGAGCTTCCAGGGGGAAACGCCTGGTATCTTTATAGTCCTGTCGGGTTTCGCCACCTCTGACTTGAGCGTCGATTTTTGTGATGCTCGTCAGGGGGGCGGAGCCTATGGAAAAACGCCAGCAACGCGGCCTTTTTACGGTTCCTGGGCTTTTGCTGGCCTTTTGCTCACATGTTCTTTCCTGCGTTATCCCCTGATTCTGTGGATAACCGTATTACCGCCTTTGAGTGAGCTGATACCGCTCGCCGCAGCCGAACGACCGAGCGCAGCGAGTCAGTGAGCGAGGAAGCGGAAG

**G**

AGCGCCCAATACGCAAACCGCCTCTCCCCGCGCGTTGGCCGATTCATTAATGCAGCTGGCACGACAGGTTTCCCGACTGGAAAGCGGGCAGTGAGCGCAACGCAATTAATGTGAGTTAGCTCACTCATTAGGCACCCCAGGCTTTACACTTTATGCTTCCGGCTCGTATGTTGTGTGGAATTGTGAGCGGATAACAATTTCACACAGGAAACAGCTATGACCATGATTACGCCAAGCTATTTAGGTGACACTATAGAATACTCAAGCTATGCATCAAGCTTGGTACCGAGCTCGGATCCACTAGTAACGGCCGCCAGTGTGCTGGAATTCGCCCTTCACCTGCCCTTCGTAAGATCTATAATTCACTGGCCGTCGTTTTACGGTACCATCGATGATGATCCAGACATGATAAGATACATTGATGAGTTTGGACAAACCACAACTAGAATGCAGTGAAAAAAATGCTTTATTTGTGAAATTTGTGATGCTATTGCTTTATTTGTAACCATTATAAGCTGCAATAAACAAGTTAACAACAACAATTGCATTCATTTTATGTTTCAGGTTCAGGGGGAGGTGTGGGAGGTTTTTTAAAGCAAGTAAAACCTCTACAAATGTGGTATGGCTGATTATGATCCTCTAGATCGAGGTCTCTGACTAAAAAAGCACCGACTCGGTGCCACTTTTTCAAGTTGATAACGGACTAGCCTTATTTAAACTTGCTATGCTGTTTCCAGCATAGCTCTTAAACCCGTAGGTGGCATCGCCCTCGCCGAACCAAGAGCTGGAGGGAGAGCTATATATACCAGGGACTTCTGGGTATGTTTTTGGGAGGTGGTGAGTGACTAAACCACTTATTCAGCTCCCTTAGATCAAGTCTGACCCTATATCATGGTGACATAAACCTGCAAACTGATAAAACCTGAAGGATCTCAAATCCAGAGTTTGTGTGAGGGATTACCGTGGTAATTTTCAAACTGTAAAGCATATGCAAAATTATCTGGTCTTGGCTTGAGTGATTGGGTGTCTCGGTGTGATGCAGGGACGTTTTCAGTGACGTGTCCTTCTCCCTCCCCCACAGGCATGCGCAGAACATTTCCCCCCTCCTTGAAGACCAGAACAAAAGACGCCGAGAGCAGGAAACTCGTCTTACTGAATGACCGAGGCTGGAGAAAGTCGACCCTAGGAGGACCTTGCAGGTGAAGGGCGAATTCTGCAGATATCCATCACACTGGCGGCCGCTCGAGCATGCATCTAGAGGGCCCAATTCGCCCTATAGTGAGTCGTATTACAATTCACTGGCCGTCGTTTTACAACGTCGTGACTGGGAAAACCCTGGCGTTACCCAACTTAATCGCCTTGCAGCACATCCCCCTTTCGCCAGCTGGCGTAATAGCGAAGAGGCCCGCACCGATCGCCCTTCCCAACAGTTGCGCAGCCTATACGTACGGCAGTTTAAGGTTTACACCTATAAAAGAGAGAGCCGTTATCGTCTGTTTGTGGATGTACAGAGTGATATTATTGACACGCCGGGGCGACGGATGGTGATCCCCCTGGCCAGTGCACGTCTGCTGTCAGATAAAGTCTCCCGTGAACTTTACCCGGTGGTGCATATCGGGGATGAAAGCTGGCGCATGATGACCACCGATATGGCCAGTGTGCCGGTCTCCGTTATCGGGGAAGAAGTGGCTGATCTCAGCCACCGCGAAAATGACATCAAAAACGCCATTAACCTGATGTTCTGGGGAATATAAATGTCAGGCATGAGATTATCAAAAAGGATCTTCACCTAGATCCTTTTCACGTAGAAAGCCAGTCCGCAGAAACGGTGCTGACCCCGGATGAATGTCAGCTACTGGGCTATCTGGACAAGGGAAAACGCAAGCGCAAAGAGAAAGCAGGTAGCTTGCAGTGGGCTTACATGGCGATAGCTAGACTGGGCGGTTTTATGGACAGCAAGCGAACCGGAATTGCCAGCTGGGGCGCCCTCTGGTAAGGTTGGGAAGCCCTGCAAAGTAAACTGGATGGCTTTCTCGCCGCCAAGGATCTGATGGCGCAGGGGATCAAGCTCTGATCAAGAGACAGGATGAGGATCGTTTCGCATGATTGAACAAGATGGATTGCACGCAGGTTCTCCGGCCGCTTGGGTGGAGAGGCTATTCGGCTATGACTGGGCACAACAGACAATCGGCTGCTCTGATGCCGCCGTGTTCCGGCTGTCAGCGCAGGGGCGCCCGGTTCTTTTTGTCAAGACCGACCTGTCCGGTGCCCTGAATGAACTGCAAGACGAGGCAGCGCGGCTATCGTGGCTGGCCACGACGGGCGTTCCTTGCGCAGCTGTGCTCGACGTTGTCACTGAAGCGGGAAGGGACTGGCTGCTATTGGGCGAAGTGCCGGGGCAGGATCTCCTGTCATCTCACCTTGCTCCTGCCGAGAAAGTATCCATCATGGCTGATGCAATGCGGCGGCTGCATACGCTTGATCCGGCTACCTGCCCATTCGACCACCAAGCGAAACATCGCATCGAGCGAGCACGTACTCGGATGGAAGCCGGTCTTGTCGATCAGGATGATCTGGACGAAGAGCATCAGGGGCTCGCGCCAGCCGAACTGTTCGCCAGGCTCAAGGCGAGCATGCCCGACGGCGAGGATCTCGTCGTGACCCATGGCGATGCCTGCTTGCCGAATATCATGGTGGAAAATGGCCGCTTTTCTGGATTCATCGACTGTGGCCGGCTGGGTGTGGCGGACCGCTATCAGGACATAGCGTTGGCTACCCGTGATATTGCTGAAGAGCTTGGCGGCGAATGGGCTGACCGCTTCCTCGTGCTTTACGGTATCGCCGCTCCCGATTCGCAGCGCATCGCCTTCTATCGCCTTCTTGACGAGTTCTTCTGAATTATTAACGCTTACAATTTCCTGATGCGGTATTTTCTCCTTACGCATCTGTGCGGTATTTCACACCGCATACAGGTGGCACTTTTCGGGGAAATGTGCGCGGAACCCCTATTTGTTTATTTTTCTAAATACATTCAAATATGTATCCGCTCATGAGACAATAACCCTGATAAATGCTTCAATAATAGCACGTGAGGAGGGCCACCATGGCCAAGTTGACCAGTGCCGTTCCGGTGCTCACCGCGCGCGACGTCGCCGGAGCGGTCGAGTTCTGGACCGACCGGCTCGGGTTCTCCCGGGACTTCGTGGAGGACGACTTCGCCGGTGTGGTCCGGGACGACGTGACCCTGTTCATCAGCGCGGTCCAGGACCAGGTGGTGCCGGACAACACCCTGGCCTGGGTGTGGGTGCGCGGCCTGGACGAGCTGTACGCCGAGTGGTCGGAGGTCGTGTCCACGAACTTCCGGGACGCCTCCGGGCCGGCCATGACCGAGATCGGCGAGCAGCCGTGGGGGCGGGAGTTCGCCCTGCGCGACCCGGCCGGCAACTGCGTGCACTTCGTGGCCGAGGAGCAGGACTGACACGTGCTAAAACTTCATTTTTAATTTAAAAGGATCTAGGTGAAGATCCTTTTTGATAATCTCATGACCAAAATCCCTTAACGTGAGTTTTCGTTCCACTGAGCGTCAGACCCCGTAGAAAAGATCAAAGGATCTTCTTGAGATCCTTTTTTTCTGCGCGTAATCTGCTGCTTGCAAACAAAAAAACCACCGCTACCAGCGGTGGTTTGTTTGCCGGATCAAGAGCTACCAACTCTTTTTCCGAAGGTAACTGGCTTCAGCAGAGCGCAGATACCAAATACTGTCCTTCTAGTGTAGCCGTAGTTAGGCCACCACTTCAAGAACTCTGTAGCACCGCCTACATACCTCGCTCTGCTAATCCTGTTACCAGTGGCTGCTGCCAGTGGCGATAAGTCGTGTCTTACCGGGTTGGACTCAAGACGATAGTTACCGGATAAGGCGCAGCGGTCGGGCTGAACGGGGGGTTCGTGCACACAGCCCAGCTTGGAGCGAACGACCTACACCGAACTGAGATACCTACAGCGTGAGCTATGAGAAAGCGCCACGCTTCCCGAAGGGAGAAAGGCGGACAGGTATCCGGTAAGCGGCAGGGTCGGAACAGGAGAGCGCACGAGGGAGCTTCCAGGGGGAAACGCCTGGTATCTTTATAGTCCTGTCGGGTTTCGCCACCTCTGACTTGAGCGTCGATTTTTGTGATGCTCGTCAGGGGGGCGGAGCCTATGGAAAAACGCCAGCAACGCGGCCTTTTTACGGTTCCTGGGCTTTTGCTGGCCTTTTGCTCACATGTTCTTTCCTGCGTTATCCCCTGATTCTGTGGATAACCGTATTACCGCCTTTGAGTGAGCTGATACCGCTCGCCGCAGCCGAACGACCGAGCGCAGCGAGTCAGTGAGCGAGGAAGCGGAAG

**Supplementary File 1: Sequences of insertion constructs purchased as dsDNA fragments.**

A: Sequence of *runx1+23* including PaqCI sites, O1 and O2. B: Sequence of *tdStayGold* including PaqCI sites, O2 and O3. C: Sequence of *LNGFR* including PaqCI sites, O2 and O3. D: Sequence of *rac2^WT^* including PaqCI sites, overhang sequence CTCG (O3B) and O4. E: Sequence of *rac2^D57N^* including PaqCI sites, O3B and O4. F: Sequence of 3E polyA-gRNA (Control):U6 including PaqCI sites, overhang sequence CGTA (O3C) and O4. G: Sequence of 3E polyA-gRNA (GFP):U6 including PaqCI sites, O3C and O4.
