# Supplementary material for: ImPaqT, a Golden Gate-based immunological toolkit for zebrafish transgenesis
Source: eLife. 2026 Jul 29;14:RP104182. doi: 10.7554/eLife.104182 (PMC13421555; doi:10.7554/eLife.104182)
Supplement: Supplementary file 2. — PaqCI sites are shown in magenta, 4-base spacer sequences are shown in blue, overhang 1 is shown in orange, O1A in purple, O1B in brown, O2 in green, O3 in pink, O3B in light green, and O4 in turquoise. Bases for mutations are highlighted in red. [file elife-104182-supp2.docx]

**ImPaqT - A Golden Gate-based Toolkit for Zebrafish Transgenesis**

Saskia Hurst, Christiane Dimmler, Mark R. Cronan

**Supplementary File 2: Primer Sequences.**

|  | **Gene** | **Primer Direction** | **Primer Sequence 5' 🡪 3'** |
| --- | --- | --- | --- |
| **5‘ Element** | *irg1* | Forward | TTTCCCCACCTGCTTCCTTCCGCAGTGAGGGGTATGGAAAA |
|  |  | Reverse | TTGTACCACCTGCTTCATTTGTGTGCGAGCTCTGAATCTTG |
|  | *ubb* | Forward | TTTCCCCACCTGCCCTTTTCCTTCTAGAATTTGTCGAAACATTTATG |
|  |  | Reverse | AAACCCCACCTGCAAGGTTTGCTGTAAACAAATTCAAAGTAAGATTAG |
|  | PaqCI mutation in *ubb* | Forward | CTTCACCT**C**CTTCCATTACAAACTATTCCCATC |
|  |  | Reverse | TGGAAGGAGGTGAAGATTTAATAATCTTACATCAAAC |
|  | *ubb_1_* | Forward | TTTCCCCACCTGCCCTTTTCCTTCTA |
|  |  | Reverse | AAACCCCACCTGCAAGGGCTATTGGTGAATCAGACTTGTGGAATCTG |
|  | *ubb_2_* | Forward | TTTCCCCACCTGCCCTTTAGCAAAGCGAATAAACAACCAAA |
|  |  | Reverse | AAACCCCACCTGCAAGGTCAAGTAAGAAAAATATCAAGCATATTAC |
|  | *ubb_3_* | Forward | TTTCCCCACCTGCCCTTTTGATCGGAAATAAGAAAAAATATAAACGT |
|  |  | Reverse | ATTATACACCTGCAAGGTTTGCTGTAAA |
|  | *mfap4* | Forward | TTTCCCCACCTGCCCTTTTCCGCGTTTCTTGGTACAGC |
|  |  | Reverse | AAACCCCACCTGCAAGGTTTGCACGATCTAAAGTCATGAAGAAAG |
|  | *mpeg1.1* | Forward | (TTTCCC)CACCTGCTTCCTTCCTTGTTGGAGCACATCTGACA |
|  |  | Reverse | (TTTCCC)CACCTGCTTCATTTGTGTTTTGCTGTCTCCTGCAC |
|  | *ubb:loxP GFP loxP* | Forward | TTTCCCCACCTGCCCTTTTCCACCAGCAAAGTTCTAGAATTTGTC |
|  |  | Reverse | AAACCCCACCTGCAAGGTTTGCGCCCTTTTTGGATCCA |
|  | *hsp70* | Forward | TTTCCCCACCTGCCCTTTTCCTCAGGGGTGTCGC |
|  |  | Reverse | AAACCCCACCTGCAAGGTTTGCAGGAAAAAAAAACAATTAGAATTAATTTTATATT |
|  | *lck* | Forward | TTTCCCCACCTGCCCTTTTCCGAATTCACAATCTCATCATCATCATCTGAGC |
|  |  | Reverse | AAACCCCACCTGCAAGGTTTGGTTTACTAAGCATGAGAGAAAATGGTGCAACTATA |
|  | PaqCI Mutation in *lck* | Forward | GCA**GG**TCCTCGTCGCTCTGAGCAG |
|  |  | Reverse | CTCAGAGCGACGAGGACCTGCTGCAA |
|  | *lyz* | Forward | TTTCCCCACCTGCCCTTTTCCCCTGATCACTGGTGTAGTGAACTC |
|  |  | Reverse | AAACCCCACCTGCAAGGTTTGTCGAGATTGTATCACTGCTGATATCTGC |
|  | *runX1+23* | Forward | purchased from Twist Bioscience, containing PaqCI recognition site, O1 and O2, full sequence in Supplementary File 1 |
|  |  | Reverse |  |
| **Middle Element** | *icre* | Forward | TTTCCCCACCTGCCCTTCAAACATG |
|  |  | Reverse | AAACCCCACCTGCAAGGTAGCGTCCCCATCCTCGAGCAGC |
|  | *tdTomato* | Forward | TTTCCCCACCTGCCCTTCAAACATGGTGAGCAAGGGC |
|  |  | Reverse | AAACCCCACCTGCAAGGTAGCTTACTTGTACAGCTCGTCCA |
|  | *tdTomato* (no stop) | Forward | (ACTGACTG)CACCTGCCCTTCAAACATGGTGAGCAAGGGC |
|  |  | Reverse | (CAGTCAGT)CACCTGCAAGGTAGCCTTGTACAGCTCGTCCATG |
|  | *tdStayGold* | na | purchased from Twist Bioscience, containing PaqCI recognition site, O2 and O3, full sequence in Supplementary File 1 |
|  |  | na |  |
|  | *nitroreductase* | Forward | TTTCCCCACCTGCCCTTCAAACATGGCCTCCGGACTCA |
|  |  | Reverse | AAACCCCACCTGCAAGGTAGCCACTTCGGTTAAGGTGATGTTTTGC |
|  | *tdTomato CAAX* | Forward | TTTCCCCACCTGCCCTTCAAACATGGTGAGCAAGGGCGAG |
|  |  | Reverse | AAACCCCACCTGCAAGGTAGCCTAGGAGAGCACACACTTGCA |
|  | *mTurquoise2* | Forward | TTTCCCCACCTGCCCTTCAAACATGGTGAGCAAGGGC |
|  |  | Reverse | AAACCCCACCTGCAAGGTAGCTTACTTGTACAGCTCGTCCA |
|  | *mNeonGreen* | Forward | TTTCCCCACCTGCCCTTCAAACATGGTGAGCAAGGGC |
|  |  | Reverse | AAACCCCACCTGCAAGGTAGCTTACTTGTACAGCTCGTCCA |
|  | *mCitrine* | Forward | TTTCCCCACCTGCCCTTCAAACATGGTGAGCAAGGGC |
|  |  | Reverse | AAACCCCACCTGCAAGGTAGCTTACTTGTACAGCTCGTCCA |
|  | *kid* | Forward | TTTCCCCACCTGCCCTTCAAAAGTCAGAATAGTGGACAG |
|  |  | Reverse | AAACCCCACCTGCAAGGTAGCCAGGAAGCGGAGCTAC |
|  | *LNGFR* | Forward | purchased from Twist Bioscience, containing PaqCI recognition site, O2 and O3, full sequence in Supplementary File 1 |
|  |  | Reverse |  |
|  | *p2A* | Forward | (ACTGACTG)CACCTGCCCTTGCTACAGGAAGCGGAGCTACTAACTT |
|  |  | Reverse | (CAGTCAGT)CACCTGCAAGGCGAGCTAGGTCCAGGGTTCTCC |
| **3‘ Element** | *p2A tdTomato* | Forward | TTTCCCCACCTGCTTCCGCTACAGGAAGCGGAGCTACTAAC |
|  |  | Reverse | TTGTACCACCTGCTTCATCCTTTACTTGTACAGCTCGTCCA |
|  | *p2A mTurquoise* | Forward | AAGGAACACCTGCAAAGGCTACAATGGTGAGCAAGGGC |
|  |  | Reverse | AAACCCCACCTGCAAGGTCCTTTACTTGTACAGCTCGTCCATG |
|  | *ubb:polyA* | Forward | TTTCCCCACCTGCCCTTGCTACAATTCTCAGTATCCCCTGC |
|  |  | Reverse | AAACCCCACCTGCAAGGTCCTATACTTCTCATTTCGCATCTTATT |
|  | *p2A dLanYFP* | Forward | TTTCCCCACCTGCCCTTGCTACAGGAAGCGGAGCTACTAACTT |
|  |  | Reverse | AAACCCCACCTGCAAGGTCCTTTACTTGTACAGCTCGTCCATG |
|  | *mTurquoise2* | Forward | TTTCCCCACCTGCCCTTGCTACAGGAAGCGGAGCTACTAACTT |
|  |  | Reverse | AAACCCCACCTGCAAGGTCCTTTACTTGTACAGCTCGTCCATG |
|  | *tdTomato* | Forward | AAGGAACACCTGCAAAGGCTACAATGGTGAGCAAGGGC |
|  |  | Reverse | AAACCCCACCTGCAAGGTCCTTTACTTGTACAGCTCGTCCATG |
|  | *mNeonGreen* | Forward | AAGGAACACCTGCAAAGGCTACAATGGTGAGCAAGGGC |
|  |  | Reverse | AAACCCCACCTGCAAGGTCCTTTACTTGTACAGCTCGTCCATG |
|  | *rac2^WT^* | Forward | purchased from IDT, containing PaqCI recognition site, O3B and O4, full sequence in Supplementary File 1 |
|  |  | Reverse |  |
|  | *rac2^D57N^* | Forward | purchased from IDT, containing PaqCI recognition site, O3B and O4, full sequence in Supplementary File 1 |
|  |  | Reverse |  |
|  | polyA-gRNA(Cntrl):U6 | Forward | purchased from IDT, containing PaqCI recognition site, O3C and O4, full sequence in Supplementary File 1 |
|  |  | Reverse |  |
|  | polyA-gRNA(GFP):U6 | Forward | purchased from IDT, containing PaqCI recognition site, O3C and O4, full sequence in Supplementary File 1 |
|  |  | Reverse |  |

**Supplementary File 2: Primer Sequences.**

PaqCI sites are shown in magenta, 4-base spacer sequences are shown in blue, overhang 1 is shown in orange, O1A in purple, O1B in brown O2 in green, O3 in pink, O3B in light green and O4 in turquoise. Bases for mutations are highlighted in red.
