# Supplementary material for: ImPaqT, a Golden Gate-based immunological toolkit for zebrafish transgenesis
Source: eLife. 2026 Jul 29;14:RP104182. doi: 10.7554/eLife.104182 (PMC13421555; doi:10.7554/eLife.104182)
Supplement: Peer review history

# Editor report: eLife Assessment

**DOI:** 10.7554/eLife.104182.3.sa0

**Authors:** Didier YR Stainier

**Affiliations:** Max Planck Institute for Heart and Lung Research Germany

**Keywords:** Convincing, Valuable

This study introduces a **valuable** toolkit for zebrafish transgenesis, significantly enhancing the flexibility and efficiency of transgene generation for immunological applications. The authors provide **convincing** evidence through well-designed experiments, demonstrating the toolkit's utility in generating diverse and functional transgenic lines.

# Referee report: Reviewer #1 (Public review)

**DOI:** 10.7554/eLife.104182.3.sa1

Summary:

The authors introduce ImPaqT, a modular toolkit for zebrafish transgenesis, utilizing the Golden Gate cloning approach with the rare-cutting enzyme PaqCI. The toolkit is designed to streamline the construction of transgenes with broad applications, particularly for immunological studies. By providing a versatile platform, the study aims to address limitations in generating plasmids for zebrafish transgenesis.

Strengths:

The ImPaqT toolkit offers a modular method for constructing transgenes tailored to specific research needs. By employing Golden Gate cloning, the system simplifies the assembly process, allowing seamless integration of multiple genetic elements while maintaining scalability for complex designs. The toolkit's utility is evident from its inclusion of a diverse range of promoters, genetic tools, and fluorescent markers, which cater to both immunological and general zebrafish research needs. Even small DNA fragements, such as the viral 2a sequence, can be cloned into a multi-component plasmid in one step. The components can be assembled from PCR fragments or synthesized DNA fragments, forgoing the need for "entry" vectors. Further, the authors show that the exisiting PaqCI sites can be domesticated to improve the versatility of the system. The validation provided in the manuscript is Convincing, demonstrating the successful generation of several functional transgenic lines. These examples highlight the toolkit's efficacy, particularly for immune-focused applications.

Comments on revisions:

The authors have addressed all the concerns raised in the first review. Congratulations to the authors for their effort.

# Referee report: Reviewer #2 (Public review)

**DOI:** 10.7554/eLife.104182.3.sa2

Summary:

Hurst et al. developed a new Tol2-based transgenesis system, ImPaqT, an Immunological toolkit for PaqCl-based Golden Gate Assembly of Tol2 Transgenes, to facilitate the production of transgenic zebrafish lines. This Golden Gate assembly-based approach relies on only a short 4-base-pair overhang sequence in the final construct, and the insertion construct and backbone vector can be assembled in a single-tube reaction using PaqCl and a ligase. This approach can also be expandable by introducing new overhang sequences while maintaining compatibility with existing ImPaqT constructs, allowing users to add fragments as needed.

The generation of several transgenic zebrafish lines for immunological studies demonstrates the feasibility of the ImPaqT in vivo. Lineage tracing of macrophages via LPS injection demonstrates the approach's functionality and validates its use in vivo.

Comments on revisions:

The authors have addressed all my concerns.

# Author comment: Author response

**DOI:** 10.7554/eLife.104182.3.sa3

**Authors:** Saskia Hurst, Christiane Dimmler, Mark R Cronan

**Affiliations:** Max Planck Institute for Infection Biology Berlin Germany; Max Planck Institute for Infection Biology Berlin Germany; Max Planck Institute for Infection Biology Berlin Germany

The following is the authors’ response to the original reviews.

We thank the reviewers and editors for their careful reading of our manuscript and thoughtful comments on it. We appreciate the overall positive opinion on our manuscript and helpful comments and suggestions from the reviewers. Overall, the main points identified by reviewers were (1) further broadening of the system to a range of inputs as well as the construct types that can be generated with the system and (2) Further consideration of any off-target joining or off-target effects on genes/proteins and the limits to the expandability of the kit. To address these concerns, we have added new data in Figure 6, illustrating the generation of a new construct using PCR and dsDNA fragments, new constructs for *mpeg1.1* and for CRISPR gRNA expression and have revised the text to further address concerns and limitations of the toolkit. We thank the reviewers and editors for these suggestions and feel that they have substantially improved the manuscript.

**Public Reviews:**

**Reviewer #1 (Public review):**

Summary:

The authors introduce ImPaqT, a modular toolkit for zebrafish transgenesis, utilizing the Golden Gate cloning approach with the rare-cutting enzyme PaqCI. The toolkit is designed to streamline the construction of transgenes with broad applications, particularly for immunological studies. By providing a versatile platform, the study aims to address limitations in generating plasmids for zebrafish transgenesis.

Strengths:

The ImPaqT toolkit offers a modular method for constructing transgenes tailored to specific research needs. By employing Golden Gate cloning, the system simplifies the assembly process, allowing seamless integration of multiple genetic elements while maintaining scalability for complex designs. The toolkit's utility is evident from its inclusion of a diverse range of promoters, genetic tools, and fluorescent markers, which cater to both immunological and general zebrafish research needs. Furthermore, the modular design ensures expandability, enabling researchers to customize constructs for diverse experimental designs. The validation provided in the manuscript is solid, demonstrating the successful generation of several functional transgenic lines. These examples highlight the toolkit's efficacy, particularly for immune-focused applications.

We appreciate the overall positive evaluation of our toolkit and the time and effort in evaluating it.

Weaknesses:

While the toolkit's technical capabilities are well-demonstrated, there are several areas where additional validation and examples could enhance its impact. One limitation is the lack of data showing whether the toolkit can be directly used for rapid cloning and testing of enhancers or promoters, particularly cloning them directly from PCR using PaqCI overhangs without needing an entry vector. Similarly, the feasibility of cloning genes directly from PCR products into the system is not demonstrated, which would significantly increase the utility for researchers working with genomic elements.

This is an excellent point. Given the increased use of gene synthesis and dsDNA fragments, we also thought it was good to demonstrate incorporation of these as well. We have added a new figure, Figure 6, which demonstrates generation of two new transgene constructs constructed by direct cloning of three PCR products along with a synthetic dsDNA fragment into a Tol2 flanked backbone plasmid as an alternative, rapid approach to generation of transgenes. The resulting plasmids, encoding the mpeg1.1. promoter, a separate p2a, and a tdTomato fluorescent protein along with either wildtype or dominant negative *rac2* were properly assembled and in transient transgenic zebrafish injected with these constructs, dominant negative *rac2* prevented macrophage recruitment to tail wounds, indicating that this approach worked for the generation of functional transgenes. These results are discussed in new text (lines 304-391) describing this new experiment and the finding that both PCR products and synthesized dsDNA could be efficiently incorporated in constructions generated with our approach as well as in the discussion (lines 494-499).

The authors discuss potential applications such as using the toolkit for tissue-specific knockout applications by assembling CRISPR/Cas9 gRNA constructs. However, they do not demonstrate the cloning of short fragments, such as gRNA sequences downstream of a U6 promoter, which would be an important proof-of-concept to validate these applications. Furthermore, while the manuscript focuses on macrophage-specific promoters, the widely used mpeg1.1 promoter is not included or tested, which limits the toolkit's appeal for researchers studying macrophages and microglia.

Yes, in the new figure described above, we have now shown that this method works with shorter PCR fragments such as the p2a fragment cloned within the tdTomato-p2a-rac2 constructs described above. This fragment is ~70 bp and while this is somewhat longer than a simple gRNA targeting sequence (though smaller than a complete sgRNA), we believe that this indicates that smaller size fragments can still be incorporated within these constructs. We also agree with the general idea of increasing functionality to incorporate CRISPR/Cas9 and now include a 3E encoding the zebrafish U6 promoter. As CRISPR expression constructs frequently incorporate complex construction, for instance, expression of tagged Cas9 along with the U6 driven gRNA as in Zhou et al., 2018 or along with rescue constructs as in Wang et al., 2021, we have given these constructs the non-standard 5’ end O3c, to enable multiplexing in these complex constructs.

We agree that it is important to include mpeg1.1, given the broad use of this promoter within the field, we’ve now included an 5E mpeg1.1 construct within the toolkit.

Another potential limitation is the handling of sequences containing PaqCI recognition sites. Although the authors discuss domestication to remove these sites, a demonstration of cloning strategies for such cases or alternative methods to address these challenges would provide practical guidance for users.

Absolutely, we have now included a new figure (Supplementary Figure 6) that illustrates one domestication approach using PCR and homology-based cloning as an easy approach to domestication. In addition, we have also mentioned alternative approaches for domestication in the discussion (lines 439-444).

**Reviewer #2 (Public review):**

Summary:

Hurst et al. developed a new Tol2-based transgenesis system ImPaqT, an Immunological toolkit for PaqCl-based Golden Gate Assembly of Tol2 Transgenes, to facilitate the production of transgenic zebrafish lines. This Golden Gate assembly-based approach relies on only a short 4-base pair overhang sequence in their final construct, and the insertion construct and backbone vector can be assembled in a single-tube reaction using PaqCl and ligase. This approach can also be expandable by introducing new overhang sequences while maintaining compatibility with existing ImPaqT constructs, allowing users to add fragments as needed.

Strengths:

The generation of several lines of transgenic zebrafish for the immunologic study demonstrates the feasibility of the ImPaqT in vivo. The lineage tracing of macrophages by LPS injection shows this approach's functionality, validating its usage in vivo.

We appreciate the positive sentiments for our toolkit and the effort put into reviewing our manuscript.

Weaknesses:

(1) There is no quantitative data analysis showing the percentage of off-target based on these 4bp overhang sequences.

While we agree that this is an important variable for the method, we feel that previous studies that have broadly tested off-target effects of all potential 4 bp overhang sequences have already given an effective overview of interactions between each of these overhangs (Potapov et al., 2018; Pryor et al., 2020). The results from these studies were incorporated into the NEB ligase fidelity viewer that we used to predict the overhangs that would have minimal off-target with each other: the tool also reports the expected off-target ligation of individual 4 bp overhangs. In all cases, we selected overhangs that would have minimal off-target efficiency, with each of the overhangs showing 1% or less off-target ligation with any of the other overhangs chosen. We have added new text, lines 119-124, that further clarifies that our selection for these ends.

(2) There is no statement for the upper limitation of the expandability.

Yes, we’ve been curious as well. While our cloning of 6 distinct fragments in Figure 5 and a new 5 fragment cloning added in revision seen in Figure 6, suggests that 5-6 fragments can be readily assembled, in the course of revisions we also attempted to generate a larger product of 11 fragments that ultimately failed. While the 11 fragment construct was unsuccessful, it is unclear whether this is due to the constructs chosen, the potential size of the plasmid or due to a failure of the technique/enzymes themselves. Given that published descriptions of PaqCI Golden Gate cloning approaches have found that PaqCI can assemble at least 32 fragments and can produce large sequences (e.g. in Sikkema et al., 2023, where they assemble the ~40 kbp T7 genome from 12, 24 and 32 distinct fragments using a PaqCI Golden Gate reaction), we suspect that our issues with the 11 fragment assembly are likely due to complications with the specific group of constructs that were combined, however, we have not been able to exhaustively test a range of constructs and assemblies of varying complexity levels. To recognize this, we have added additional text (lines 490-493) to the discussion describing that we have only combined 6 constructs, but that we think that this likely encompasses many of the applications that may be needed for this system, while recognizing that expansion beyond this number may be possible.

(3) There is no data about any potential side effect on their endogenous function of promoter/protein of interest with the ImPaqT method.

Absolutely, we have added new text (lines 457-470) to our discussion describing the potential side effects on protein function. For instance, the need to be aware of whether N- or C-termini of proteins can be modified and recognition of the potential for affecting/creating ectopic transcription factor binding sites as potential pitfalls to keep in mind.

**Recommendations for the authors:**

**Reviewer #1 (Recommendations for the authors):**

The data presented in the manuscript is robust and well-supported. However, to fully demonstrate the broad applicability of the toolkit and strengthen its impact, a few additional experiments could be beneficial. Specific suggestions for these experiments and areas of improvement are outlined in the 'Weaknesses' section of the Public Review. Additionally, Figures 2-4 illustrate the same concept - cloning three fragments from entry vectors-which comes across as repetitive. Incorporating a more diverse range of use cases would better highlight the versatility of the toolkit.

As we described in our replies to your public points above, we have now added new Figure 6 and new Supplementary Figure 6 addressing the cloning of PCR fragments, short fragments as well as a mechanism of domestication. We have also included the *mpeg1.1* promoter within the toolkit. In addition, your point on the repetition of assay is fair and in our new Figure 6, we instead used wild type and dominant-negative *Rac2* expression and failure of macrophage recruitment to the tail wound.

**Reviewer #2 (Recommendations for the authors):**

Hurst et al. developed a new Tol2-based transgenesis system ImPaqT, it is interesting and potentially efficient, but I have a few concerns:

(1) The author claimed that the ImPaqT system is more efficient than other existing systems. The authors should provide such data to support their claim.

Our argument wouldn’t be that the ImPaqT system is strictly speaking more efficient, but rather that the combination of minimal added sequence, the ability to expand or contract the fragments used, and, in our new Figure 6, the ability to directly utilize PCR products and dsDNA fragments, while retaining the ability to combinatorially build constructs from a suite of existing sequences is the main point of the method. We now explicitly state that Golden Gate cloning isn’t more efficient than existing techniques in the text (lines 534-537), but rather the particular strength of the method is the flexibility and minimal added sequence.

(2) The ImPaqT is theoretically less prone to have off-target effects than existing systems, the authors should provide such data to validate their claim.

Good point, we have now searched the zebrafish genome for PaqCI sites as well as for BsaI and BsmBI which are the 6-base cutters most commonly used for Golden Gate cloning. We found that PaqCI cuts every ~17 kb in the zebrafish genome while BsaI and BsmBI cut every ~9 kb or ~13 kb respectively, further supporting that PaqCI sites are rarer in the genome and should generally require domestication less often. We have now added new text describing this in lines 129-132.

(3) The authors should mention any potential side effects of this system on the endogenous function of the promoter/protein of interest, at least in their discussion part.

Yes, this should absolutely be expanded, as we said in your public comments above, we have now added new text describing potential pitfalls that this method may have on promoter or gene expression.

(4) The authors are suggested to provide a balanced discussion about the expandable usage of this system beyond the immune system.

We agree, this is also a good point that we should have emphasized more. We’ve added new text (lines 537-541) recognizing that in principle, many of the components we’ve derived should be useful in non-immune systems, but we also recognize that adapting this to new tissues will require the development of new promoters within the Golden Gate system which can be combined with these already developed tools.

References

Potapov, V., Ong, J.L., Kucera, R.B., Langhorst, B.W., Bilotti, K., Pryor, J.M., Cantor, E.J., Canton, B., Knight, T.F., Evans, T.C., Jr., et al. (2018). Comprehensive Profiling of Four Base Overhang Ligation Fidelity by T4 DNA Ligase and Application to DNA Assembly. ACS Synth Biol 7, 2665-2674.

Pryor, J.M., Potapov, V., Kucera, R.B., Bilotti, K., Cantor, E.J., and Lohman, G.J.S. (2020). Enabling one-pot Golden Gate assemblies of unprecedented complexity using data-optimized assembly design. PLoS One 15, e0238592.

Sikkema, A.P., Tabatabaei, S.K., Lee, Y.J., Lund, S., and Lohman, G.J.S. (2023). High-Complexity One-Pot Golden Gate Assembly. Curr Protoc 3, e882.

Wang, Y., Hsu, A.Y., Walton, E.M., Park, S.J., Syahirah, R., Wang, T., Zhou, W., Ding, C., Lemke, A.P., Zhang, G., et al. (2021). A robust and flexible CRISPR/Cas9-based system for neutrophilspecific gene inactivation in zebrafish. J Cell Sci 134.

Zhou, W., Cao, L., Jeffries, J., Zhu, X., Staiger, C.J., and Deng, Q. (2018). Neutrophil-specific knockout demonstrates a role for mitochondria in regulating neutrophil motility in zebrafish. Dis Model Mech 11.
